# Sensitivity study of a locally developed six electrode focused impedance method

**DOI:** 10.2478/joeb-2024-0005

**Published:** 2024-04-24

**Authors:** Trilochan Khanal, K Siddique-e Rabbani

**Affiliations:** 1 Department of Physics, Tribhuvan University, Kirtipur, Nepal; 2 Department of Biomedical Physics & Technology, University of Dhaka, Dhaka, Bangladesh

**Keywords:** Electrical bioimpedance, FIM, focused impedance, sensitivity study, indigenous technology

## Abstract

The Focused Impedance Method (FIM) is a new technique of electrical bioimpedance measurements in the human body. The idea originated in Bangladesh and provides an opportunity for localized measurement of bioimpedance down to reasonable depths from the body surface using skin surface electrodes. This has potential applications for physiological studies of targeted organs in the body and in detecting or diagnosing diseases and disorders. FIM is based on the age-old Tetra-Polar Impedance Measurement (TPIM) but provides a few significant improvements.

Technology must be developed indigenously to obtain long-term benefits, particularly in Low and Medium Income countries (LMIC). This paper presents an experimental sensitivity study of the six-electrode version of the Focused Impedance method (FIM-6) with the circuit and phantom indigenously designed in Nepal. The work involved sensitivity studies of both FIM-6 and TPIM with the necessary circuit blocks developed through experimental validation. The sensitivity studies were performed on a simple 2D phantom with different electrode arrangements for FIM-6 and linear TPIM. A cylindrical object was placed at different positions for this study. The FIM-6 gave a high sensitivity in the central part, which remained almost constant within a small region that may be termed as the focused region. On the other hand, TPIM results fell off sharply away from the central point, making it unsuitable for practical measurements on target organs. Besides, there were areas with large negative sensitivities in TPIM, which were much smaller in FIM. The results obtained through this work clearly show the improvement offered by FIM over TPIM.

## Introduction

Recording bioelectric signals from the body to assess physiological disorders has been a subject of research for many decades. Different approaches and electrode configurations were employed to measure the impedance of the human body, which is essentially a volume conductor. A basic measurement, called the bipolar measurement technique, uses two electrodes through which an alternating current is passed while the potential developed across the same pair of electrodes is measured. However, the high value of contact impedances of the electrodes makes it difficult to measure small variations of internal body impedance which are the target organs in most of such measurements. To eliminate or minimize the effect of the contact impedances, Tetra-polar Impedance Measurement Systems (TPIM) were introduced [1] and are widely used.

**Figure 1:**
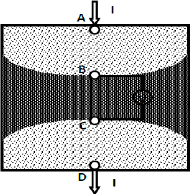
Tetra-polar impedance measurement scheme [3].

In a typical measurement set up for TPIM, as shown in Figure 1, a constant current is injected through a pair of current electrodes (‘A’ and ‘D’) and the resulting potential ‘V’ is measured through another pair of potential electrodes (‘B’ and ‘C’). The ratio of measured voltage to applied current is called Transfer Impedance [2].

The extent to which the contribution of a subvolume within a volume conductor contributes to the measured transfer impedance in a homogenous and uniform conductor, is called the sensitivity of that particular subvolume. The zone of positive sensitivity is the sensitivity of the region between the potential electrodes in the volume conductor (as shown by the dark-shaded region in Figure 1), such that increased impedance within the region increases the resulting transfer impedance. Zones of negative sensitivity exist between the current and potential electrodes (the lightly shaded zones in Figure 1), which negatively contribute to the transfer impedance; meaning that increased impedance within these regions will decrease the resulting transfer impedance [2]. This jeopardizes the measurement of impedance values of the desired region and has to be kept in purview when using TPIM.

A major improvement in the bioimpedance measurement was the introduction of Electrical Impedance Tomography (EIT) by a group in Sheffield, UK. EIT is an imaging technique to map the conductivity distribution of the body tissue essentially in a 2D plane using 16, 32 or more electrodes around the cross-section [4]. However, the current in EIT is not confined to 2D only and hence the region of interest is not localized [4]. Efforts to create a 3D volume image using EIT have also been going on for some time [5]. However, EIT is very complex and potentially expensive, particularly given applications in low and medium-income countries (LMIC).

**Figure 2:**
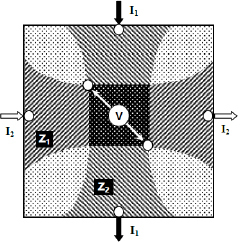
Basic concept of FIM-6 [3].

As a bridge between TPIM and EIT, the Focused Impedance Method (FIM) was conceived and developed at Dhaka University of Bangladesh, which uses 8, 6 or 4 electrodes in its three versions to localize a small zone in a volume conductor [6,7]. Here the four-electrode version is different from conventional TPIM. The 6-electrode FIM configuration (FIM-6) is shown in Figure 2 for a two-dimensional conductor. Here, small white circles indicate electrodes. Two alternating currents, I_1_ and I_2_ that are in phase but electrically isolated are driven between two electrode pairs arranged vertically and horizontally, respectively as shown in the Figure. Two electrodes for measuring potential (potential electrodes) are placed at intermediate diagonal positions, which are the intersection points of respective equipotential lines due to the two currents. The potentials V_1_ and V_2_ across these potential electrodes resulting due to current I1 and I2 respectively, give the potential difference between the two equipotential lines as shown by the edges of the horizontally shaded region and vertically shaded region, respectively. Furthermore, the values V_1_/I_1_and V_2_/I_2_ essentially give the Transfer Impedance Z_1_ and Z_2_ of the horizontal shaded region and vertical shaded region, respectively, though the contribution of different points within this region will vary.

Now, if the two currents (I_1_ and I_2_) are electrically isolated, keeping their magnitude and frequency the same and with the same phase (coherent), then the measured potential will essentially give a summation of the two transfer impedances Z_1_ + Z_2_, which will have enhanced contribution from the dark shaded region in the center. This sum was initially termed as Focused Impedance by the Dhaka University group. Later, an algebraic average of the two impedance values was termed as the Focused Impedance for a better representation.

TPIM and FIM are techniques suitable for healthcare in low and medium countries like Nepal. However, to ensure that most of the population gets the benefits of these modern technologies, local capacity development is essential. Every country or region has special circumstances in terms of the availability of raw materials, expertise, and industrial infrastructure, based on which local improvisation is needed. Although much of the basic work on these techniques has been performed elsewhere, it needs to be done in Nepal under its existing conditions. This was the motivation behind the present work. The circuit required for the measurement was designed indigenously using locally available electronic components [9]. The design process also incorporated the use of two ferrite transformers which were also designed in the lab here in Nepal, which was a challenge. Different parameters of the designed circuit were tested carefully before implementing it in the actual measurements.

This paper presents the sensitivity study using the 6-electrode version of FIM with the circuit and a measurement phantom designed indigenously in Nepal and described in a previous publication [9]. Although in a volume conductor, TPIM and FIM have 3D distribution patterns of sensitivity, this present work is targeted to study essentially the 2D sensitivity patterns.

## Materials and methods

### Basic analysis

For a TPIM arrangement, if the amplitude of the driven constant current is I and if V is the potential measured between the two central electrodes, then the Transfer Impedance is,

(1) Z=V/I

The term ‘Transfer’ is used to differentiate this from a conventional two-electrode system where current and potential electrodes are the same [10]. However, the terms ‘Transfer Impedance’ and ‘Impedance’ will be used interchangeably in this paper since the present work does not involve two electrode measurements.

Since the current I is constant, therefore,

(2) Z∝V

This means the measured voltage directly gives a measure of the impedance, and while comparing relative measurements, using potential values only should suffice.

For a FIM-6 arrangement, the Transfer Impedance is essentially the average of the two impedances obtained using the two concentric and orthogonal TPIM arrangements. Thus, if the two transfer impedances are Z_1_ and Z_2_, then FTZ, the focused transfer impedance obtained using the FIM configuration would be given by,

(3) $$\text{FTZ}=\left({\text{Z}}_{1}+{\text{Z}}_{2}\right)/2$$

However, the measurement of the potential between the two diagonal electrodes at the center essentially adds the two TPIM values, therefore, this essentially gives twice the above value, i.e., 2FTZ. However, as mentioned before, for relative measurements it does not matter whether one uses FTZ or 2FTZ, if consistency is maintained.

### Circuit arrangement for the study

The circuitry developed for FIM-6 and described in a previous publication [9] was used. It essentially provides coherent sinusoidal alternating currents with the same current amplitude (constant current) through two orthogonal current drive electrode pairs shown in Figure 2. The potential developed across the diagonal electrodes in the central region was measured using a differential amplifier which is proportional to FIM impedance, since current is constant. The frequency of the alternating current used was 10 kHz.

### Phantom Design

**Figure 3:**
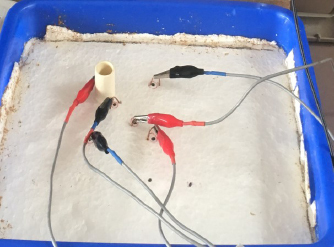
Photograph of the phantom used in the study. Tap water of about 1cm height covers the base made with a cork sheet (foam plastic). The plastic pipe shown is an insulating object which was placed at different points to measure the sensitivity of the system.

The present work essentially studies the 2D sensitivity of FIM-6. For comparison, TPIM was also studied using the same phantom. An experimental phantom was designed simply using a rectangular plastic tray, 28 cm x 23 cm, into which a thick cork sheet (foam plastic) was pushed in tight to form the base, as shown in Figure 3. The cork sheet of about 1.5 cm thickness and cut exactly in the shape of rectangular phantom, was tightly fixed on the base using Fevicol mixed with saw dust. Also, the cork sheet was sealed sideways to avoid any water leaking sideways, as shown in the figure.

For the electrodes, metallic rods with a cylindrical cross-section would have been ideal providing geometrical symmetry. However, to make a low-cost experimental arrangement we used a base of soft polystyrene foam (cork sheet) and an inserted rod will tilt easily when connected using a crocodile clip. Therefore, we used a U-shaped thick copper wire, which when inserted, gives a better mechanical strength in maintaining its configuration. Effectively it would form a wide electrode, but since the width is small compared to the dimensions of the whole water tray, it would still give acceptable measurements to evaluate the performance expected of a focused impedance method.

**Figure 4:**
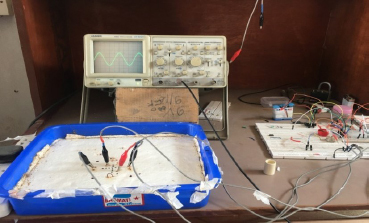
A laboratory set up for Phantom study.

Tap water about a cm deep above the base was then added to it, which is assumed to make an essentially 2D phantom for this study. The electrodes were then connected using crocodile clips for the measurements. A piece of a cylindrical plastic pipe of 2 cm diameter, which acted as a circular insulating object in 2D, was placed at different points on the base for the sensitivity study. The potential output was measured using an oscilloscope. Figure 4 shows the whole laboratory set-up.

**Figure 5:**
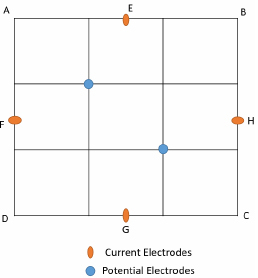
Placement of electrodes for FIM-6 phantom study.

The measurements for FIM-6 are explained with the help of Figure 5. Here ABCD represents a square region with 6 cm sides within the 28 cm x 23 cm phantom. It is divided into 9 equal square zones in a matrix, each cell with 2 cm sides. The electrodes were affixed with reference to this square region.

A constant alternating current was driven through the electrodes F and H (shown red) in the horizontal direction while another constant alternating current of the same magnitude and phase but electrically isolated from the above, was driven between electrodes E and G (shown red) in the vertical direction. Potential developed across a pair of diagonal electrodes (shown blue) placed at the corners of the mid-square cell was measured which essentially gives the impedance value of the FIM-6 as a sum, and which essentially equals 2FTZ. One may divide it by 2 to get an average. However, since the change in the values is of concern due to a change in a localized change in the volume conductor, the above two options will essentially give the same result.

Readings were taken for FTZ with only tap water (background) and placing the plastic pipe at several chosen points along both horizontal and vertical directions through the center.

The same set up was used to measure TPIM using one pair of current drive electrodes. For this, electrodes F and H were used to inject a constant current while the blue electrodes at the corners of the mid-square region were used to measure potential. Readings were noted for the TPIM technique by placing the plastic pipe in the phantom at several positions, some of which went beyond the boundary of the square matrix shown at the center.

To maintain consistency in the placement of the center of the circular cross section of the plastic pipe, the chosen points were marked on the plastic base using a marker pen.

**Figure 6:**
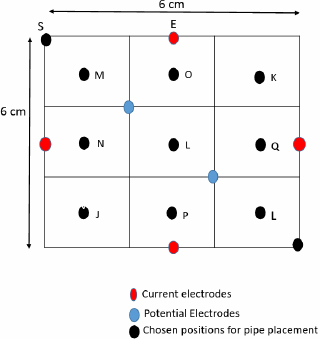
Positions for object placement for TPIM and FIM-6 phantom study.

### Ethical approval

The conducted research is not related to either human or animal use.

## Results

### Linear TPIM Phantom Study

The results of the TPIM (for the horizontal electrode arrangement) are presented first. The current peak of the driven constant current was 0.27 mA, as measured. The transfer impedance of the background, without the plastic pipe, was measured to be 875 ohms.

**Figure 7:**
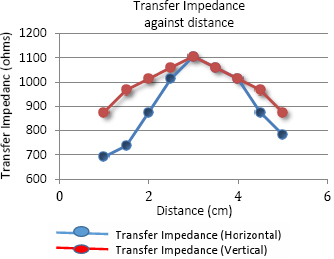
Transfer Impedance against distance for linear TPIM for the measurement along horizontal and vertical directions.

The graph in Figure 7 shows the variation of transfer impedance with the plastic pipe (hereafter referred to as the object) placed at the center and at eight chosen positions along the horizontal and the vertical directions. The graph shows that the maximum value of transfer impedance at the center is about 1106 ohms, which is expected since the object is an insulator. The value of transfer impedance decreases away from the center for both the directions of object placement. Along the vertical direction the lowest value of transfer impedance was 875 ohms with the object placed at both edges, E and G, which equals the transfer impedance value of the background. That means that because of the distance, the sensitivity of the object was too low and no effect due to the object was observed.

However, for the horizontal direction, the measured values went even below the background when the object was placed near the edges. These are the positions between the current and potential electrodes, and TPIM has negative sensitivities in these regions, an important disadvantage of the simple TPIM technique.

**Figure 8:**
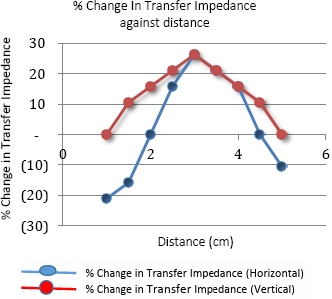
Percentage change in Transfer Impedance against distance for linear TPIM along and perpendicular direction of electrode arrangement.

The graph in Figure 8 shows the variation of percentage change in transfer impedance, derived from the values shown in Figure 7, with respect to the background impedance value. It shows a change of +26% at the center, going down to zero in the vertical direction at the two edges. On the other hand, for the horizontal direction, it clearly shows the negative changes at the edges, which were −21% and −11% respectively.

**Figure 9:**
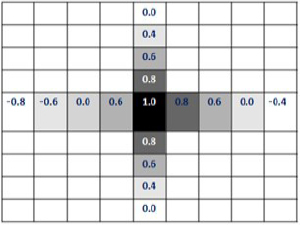
Matrix Representations of Normalized Changes of Transfer Impedance for Linear TPIM.

The normalized percent changes in impedance along the two directions through the center are shown in matrix form in Figure 9, with the maximum change at the center of the symmetry being taken as 1. The degree of darkness of the matrix elements indicates approximately the resulting change in impedance in the corresponding matrix positions.

Figure 9 depicts some matrix elements with considerable negative values, (0.8) and (0.4) near the left and right edges respectively. These points lie very close to the position of the current electrodes F and H, respectively, as shown in Figure 5.

### FIM-6 phantom study

**Figure 10:**
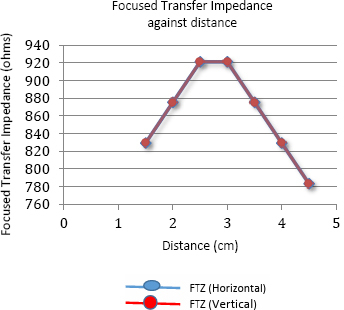
Focused Transfer Impedance against a distance measured along both horizontal and vertical directions.

**Figure 11:**
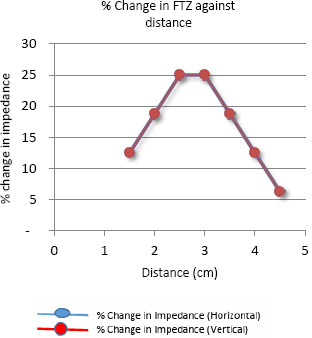
Percentage Change in FTZ against distance for Horizontal and vertical direction of measurement

The graph in Figure 10 shows the variation in focused transfer impedance (FTZ) with the insulating object placed at various positions between the points F and H along the horizontal direction and between the points E and G along the vertical direction for the set up as shown in Figure 5. The two graphs coincide exactly, so only one line graph is observed. The graph in Figure 11 illustrates the corresponding percentage variation in impedance. No negative sensitivity points were observed during the measurements along both these directions.

The small asymmetry observed during individual measurements on both sides of the center of the arrangement is due to the non-symmetric electrodes used in the phantom. Besides, the phantom used in the measurement is not square-shaped, which also contributed to the asymmetry. The measurements show that the values of focused transfer impedance (FTZ) and their corresponding percentage changes are maximum when the object was placed in between the potential electrodes while these values are much lower when the object was placed near the current electrodes.

**Figure 12:**
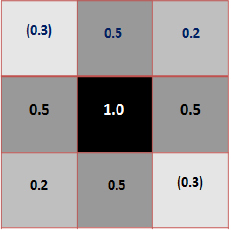
Matrix Representation of Normalized Values for FIM-6 arrangements measured along chosen positions.

The matrix representation of the results is shown in Figure 12 in terms of normalized changes in both numerals and color to give a qualitative picture. The maximum value of 1.0 is represented at the center with dark color. Although there are no points along the vertical and horizontal directions through the center, points with negative sensitivity were observed along one of the diagonal directions with a negative value of 0.3 (shown in bracket).

### Comparative Study of FIM-6 and Linear TPIM

The results obtained for both FIM-6 and linear TPIM derived from FIM-6 measured along both horizontal and vertical directions, are compared in the following graphs.

**Figure 13:**
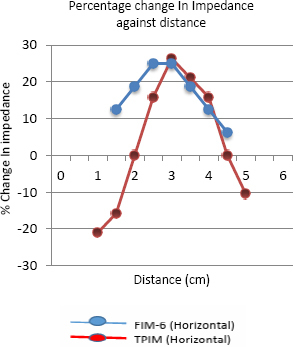
Percentage change in Impedance against distance for the Horizontal measurement of TPIM and FIM-6 configuration.

Figure 13 illustrates the variation of percentage change in impedance with the position of the center of the insulated object (pipe) along the horizontal direction for both the FIM-6 and TPIM arrangements. It shows that the maximum percentage change in focused transfer impedance at a distance of 3 cm, which is the center of the electrode arrangement. No points with negative sensitivity were observed for the FIM-6 arrangement. On the other hand, points with -21% and -11% values were observed for Linear TPIM, near the current electrodes F and H, respectively (Figure 5). The Figure suggests that TPIM is very “sharp” in the middle, so any small variation in the placement of the object will change measurements significantly. On the other hand, the variation is less in FIM, so small variations in object placement will not significantly change the measurements, which is another advantage of FIM.

**Figure 14:**
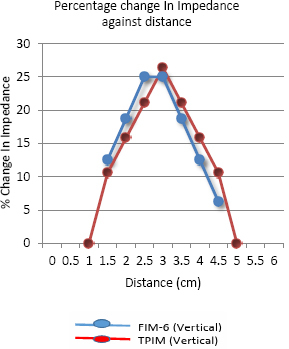
Percentage change in Impedance against a distance measured for the perpendicular direction of the electrode arrangement of TPIM and FIM-6 configuration.

The graph in Figure 14 shows the percentage change in transfer impedance against the distance of measurement points for both TPIM and FIM-6 configurations measured along the vertical direction with respect to Figure 6 (current drive for TPIM is along the horizontal direction). Again, the maximum percentage change in impedance is found at a distance of 2.5 and 3 cm from the edge of the electrode arrangement for the FIM-6 arrangement. On the other hand, the maximum value for percentage change in transfer impedance for Linear TPIM arrangement is at a distance of 3 cm with a value that is very close to that of the FIM-6 arrangement. However, the TPIM values drop down to zero on both sides sharply, but there appear no points with negative sensitivity.

## Discussion

Although much work has already been done on FIM by the innovating group in Dhaka and elsewhere [11], the main motivation of the present work was to design and develop the whole instrumentation locally in Nepal and evaluation of the system using a simple experimental phantom, again designed, and developed locally in Nepal. The instrumentation developed and tested gave satisfactory performance and was published earlier [9].

The specific focus of the present work was to design a phantom and perform measurements to understand the sensitivities of TPIM and FIM in two dimensions using the locally developed equipment. The two-dimensional phantom was designed using locally available materials in a very simple way. The focusing effect of the present six-electrode FIM has been studied simultaneously with the conventional four-electrode TPIM system, to compare the results.

The phantom studies using configurations as shown in Figures 5 and 6, show that for the 2 cm object (insulating pipe) the maximum change in the focused transfer impedance was about 25% at the center of the focused zone, which was almost the same for TPIM. The values fall off on both sides for both the configurations. However, for TPIM, the fall is sharp just around the central maximum while it is not so sharp for FIM. This suggests that any small variation in electrode or object position near the center will change measurements significantly in TPIM, while in FIM, the variation is less, so such small variations in measurement conditions will not significantly change the measurements. This is an advantage in practical application of FIM for clinical applications.

Previous studies suggest that even for FIM there are points with negative sensitivity in between current and potential electrodes [6]. However, in this work, since a large object (a pipe with 2 cm diameter) was used in a measurement area of 6 cm x 6 cm, as defined by electrode placement, negative sensitivities are expected to be less than that of point sensitivities. However, as can be seen from the results, the magnitudes of negative values obtained with FIM are much smaller with respect to that obtained using TPIM. This is a big advantage of FIM over TPIM.

Although both TPIM and FIM give high values at the center which fall off with distance of the object in both the directions measured (vertical and horizontal through the center), TPIM falls off sharply away from the central point, while FIM is almost constant over a small region and then falls off. This is again an advantage of FIM since in practical situations, one cannot place objects of interest exactly at the center of the electrode arrangement. Therefore, even for small variations of object position around the center, FIM value will not change greatly while TPIM will change greatly.

The small asymmetric results obtained for both FIM-6 and Linear TPIM could be improved using symmetric electrodes and employing the square-shaped phantom rather than the rectangular one. To make the electrodes symmetric, round pins and a single pin for each electrode could be used.

FIM offers a noninvasive and low-cost indigenous technology which can benefit the people who are deprived of modern health care technology. The sole motive of this work is to develop the FIM technique indigenously. This paper presents the sensitivity study of the technique in comparison with the conventional TPIM technique, which validates that the designed circuit is working well and could be used for physiological studies in a human body. The designed circuit for FIM-6 will first be incorporated for lung ventilation studies in the human body. Later, the study will be extended to other physiological areas.
